# The Passage of Years: Not a Matter of Covert Retrieval of Autobiographical Memories

**DOI:** 10.3389/fpsyg.2021.744551

**Published:** 2021-10-14

**Authors:** Ferdinand Kosak, Sven Hilbert

**Affiliations:** Department of Psychology, University of Regensburg, Regensburg, Germany

**Keywords:** passage of time judgment, subjective experience of time, contextual change hypothesis, storage size metaphor, autobiographical memory, covert retrieval

## Abstract

In current research, variations in retrospective passage of time judgments for long intervals are commonly attributed to differences regarding the number of experiences in these intervals or the accessibility of the respective memories. This seems to imply the assumption of a covert retrieval, where authors presume that memories from the respective interval influence the experience of time without these memories being explicitly activated when judging. However, no studies have systematically investigated the relation between the experience of time and the respective experiences and memories. To this end, we analyzed data from three studies in which participants judged the passage of the last 5 years either before being asked to select outstanding life events from a list (Studies 1a and b; *N* = 293 and 263) or before recalling as many meaningful personal memories as were spontaneously accessible (Study 2; *N* = 262). Despite applying a statistically powerful trial-by-trial mixed-effects modeling approach, neither in the separate datasets nor in the combined dataset, passage of time judgments were predicted by the number of reported events or memories. This suggests that people's spontaneous judgments of the passage of multiannual intervals are not necessarily affected by a covert retrieval of memories from the respective period.

## Introduction

The velocity with which humans perceive time to pass by is a common matter of discussion in both daily life, where people often seem to complain about time flying, as well as in academics, where researchers try to validate and explain this phenomenon. In fact, when asking participants about their experience of time passing for intervals ranging from days up to a decade, responses predominantly indicate an experience of time passing rather fast (Flaherty and Meer, 1994; Wittmann and Lehnhoff, 2005; Friedman and Janssen, 2010). In attempting to explain the subjective experience of time, different theoretical and empirical approaches have emerged.

### Ratio-Theory

**Figure 1:**
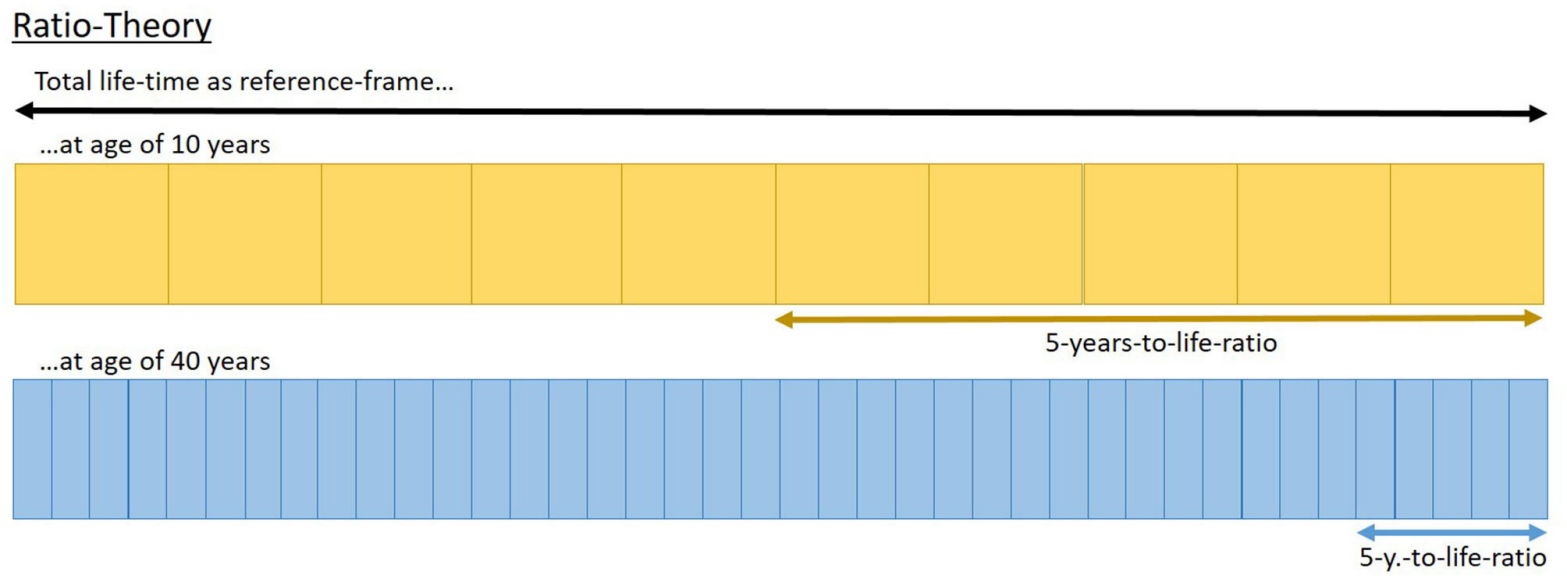
Illustration of ratio-theory suggesting that perception of time-units is affected by the relative length of these units compared to life-time. This depicts that an interval of 5 years covers 50% of the life-time from a 10 year old person but only 12.5% of the life-time from a 40 year old person.

Already in the 19th century, Janet (1877) and James (1863; as cited in Block et al., 1998) brought up the idea of a ratio-theory: given that the ratio of time units to life gets smaller with every unit, they argued that the relative length of these units compared to our lives constantly decreases (see Figure 1). Assuming that our perception of duration has to be in relation to some reference, and considering life as a whole as a potential all-embracing reference frame, the process of aging could evoke the impression of time passing faster than in the past at every single point in our lives. Implying that humans set a prototypical concept of the felt duration/velocity of the established time units early in their lives, this still seems a plausible and valuable approach to explain why time seems to pass fast in adulthood. An approach, however, that is hardly accessible for empirical research and consequently lacks such support.

### Internal-Clock Models

**Figure 2:**
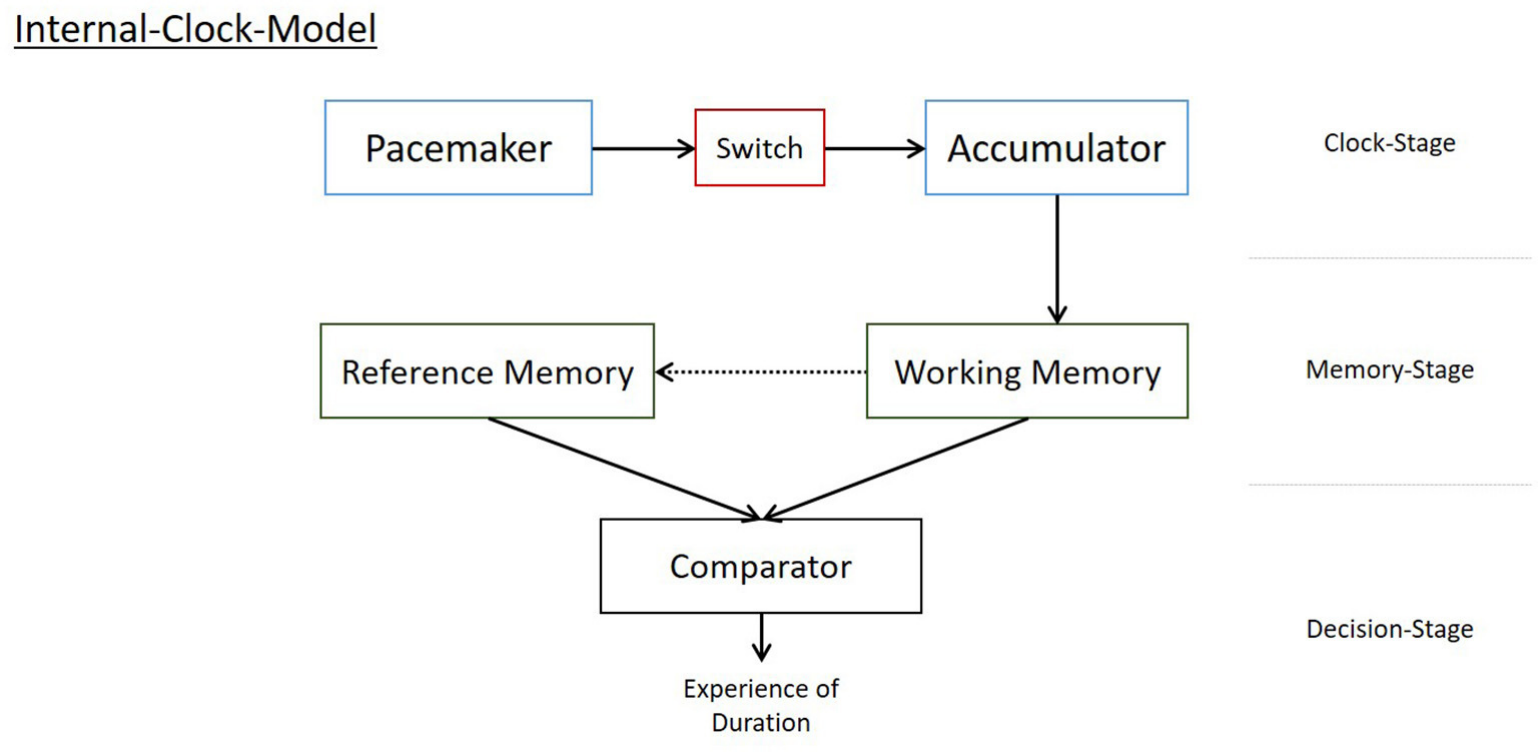
Illustrative example of an internal clock model. Note that many versions with minor and major differences have been suggested, e.g., by Treisman (1963), Gibbon et al. (1984), Zakay and Block (1995), or Droit-Volet and Meck (2007). This depiction tries to capture the most common components without arguing in favor of or against any of the models suggested. For a detailed discussion regarding the development of internal-clock-models see Wearden (2016).

By contrast, the idea of an internal clock has become one cornerstone of modern empirical studies on time-perception. These models imply a process in which pulses are produced, stored in working memory, and compared to a reference memory. The pulses serve as time-units and the comparison to protoypical time-units retrieved from a reference memory results in an experience of duration (see Figure 2). These models have proven valuable for explaining the experience of time in short invervals (ranging from ms to s), in particular for prospective timing (i.e., where participants are aware of their experience of time being the matter of the investigation; e.g., Droit-Volet and Meck, 2007). However, they are hardly used to explain retrospective time perception (i.e., where participants report their time perception after the interval in question and without knowing about time as matter of investigation during the interval; for a comprehensive view of different paradigms used in psychological research, see Kosak and Kuhbandner, 2021). Robust findings show a slowing down of the internal clock with aging, indicated, for instance, by increasingly longer productions of given intervals (e.g., Craik and Hay, 1999; Espinosa-Fernández et al., 2003). This is sometimes discussed as a possible explanation for time speeding up with age. However, this interpretation seems inplausible as evidence shows that passage of time judgments (POTJs, i.e., the velocity of time having passed; for an introduction to POTJs, see Wearden, 2005 or Wearden, 2016) differ between older and younger adults only for intervals of 5 years and longer, but not for a variety of intervals ranging from 1 h up to 1 year (e.g., Wittmann and Lehnhoff, 2005; Friedman and Janssen, 2010; Kosak et al., 2019). This suggests that the velocity with which we perceive long intervals to pass by is a different phenomenon than the estimation of duration for short intervals (see also Droit-Volet and Wearden, 2016, for an empirical approach showing that duration judgments, explicable by internal-clock models, are often incompatible with POTJs for the same intervals).

### Time-Pressure

Thus, most work covering retrospective passage of time judgments relates in one way or another to memories. In recent years, for example, some studies have investigated the relation between passage of time judgments and perceived time pressure (Friedman and Janssen, 2010; Janssen, 2017). These studies were based on the idea that the perception of time pressure leads to the impression of time having passed fast (Janssen, 2017). Although the exact mechanisms linking perceived time pressure to the experience of time passing fast could benefit from a more detailed explanation, a set of studies supports this association in general (e.g., Friedman and Janssen, 2010; Janssen et al., 2013). It seems particularly interesting that participants of all cohorts perceive high levels of time pressure at the present time while recalling to having had less time pressure in the past (Janssen, 2017). This suggests a possible explanation for the phenomenon that adults across different age groups report time to be passing fast: when currently perceiving high levels of time pressure while—at the same time—being under the (presumably) wrong impression of having had less time pressure in the past, the present perception of time passing fast might be nothing but an illusion resulting from the fading of memories from the past (Janssen, 2017). In this case, particularly memories of the experience of time pressure.

### Telescoping

**Figure 3:**
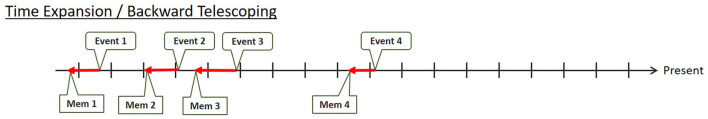
Illustration of Time Expansion due to backward telescoping of events from the remote past. Event-markers (“Event”) illustrate the factual date of an event while memories of these events (“Mem”) might be systematically dated back too far. The time between now and the wrongly remembered date is therefore expanded.

The misdating of memories, discussed as telescoping, is another theoretical approach that has repeatedly gained attention from researchers investigating the passage of time. Telescoping happens in two directions: While forward telescoping describes the tendency of dating past events too close to the present, backward telescoping describes the opposite effect, that is, dating past events too far to the past (e.g., El Haj et al., 2017). Forward telescoping has been shown to happen in particular for events from the remote past, while there is an overall tendency of backward telescoping for recent events (Thompson et al., 1988; Janssen et al., 2006; El Haj et al., 2017). The latter has also been discussed as time expansion (see Figure 3), emphasizing that this dating error potentially has impact on the experience of time: Crawley and Pring (2000) found that older people generally date events farther to the past than younger people. They interpret this decrease in time expansion as a potential mechanism explaining the perceived acceleration with aging. They seem to suggest that older people's systematic impression of past events being more distant compared to when they were younger might leave them under the impression of subjectively more time having passed than actually did. However, no studies have systematically investigated the interplay of telescoping with any measure of the subjective experience of time.

### Storage-Size and Contextual-Change Hypothesis

When trying to explain the retrospective experience of time, the most established theoretical approaches highlight the importance of memories that are retrievable from the respective time-intervals. Ornstein's work (Ornstein, 1969) is often mentioned as having provided fundamental theory and research for this account. He suggested that the sum of content stored from an interval, resulting from the number of events encoded as well as from the complexity of the encoding-process (investigated by using auditive and visual materials), is decisive for the experience of duration of the respective interval. Ornstein himself considered his model as tentative and a starting point for further research. Consequently, other researchers, in particular Block (1974, 1978), and Block and Reed (1978), showed limitations of Ornstein's theory in a number of experiments. For example, they presented evidence showing that manipulating the level of processing only (shallow vs. deep) did not necessarily lead to longer duration judgments despite deep processing in fact leading to more information being stored. Furthermore, they reported findings depicting the so-called positive time-order-error (the same interval is perceived as shorter when experienced twice), which they claimed to be inexplicable using Ornstein's Storage-Size-Metaphor. They concluded that not all information is equally relevant but that “the remembered amount of change in cognitive context during an interval” is the key-variable in explaining perceived duration of these intervals (Block and Reed, 1978, p. 657).

The development of the contextual-change-hypothesis is based on work investigating retrospective duration judgments for short intervals ranging from seconds to minutes, often operationalized by indicating duration on lines in relation to reference-intervals (e.g., Block, 1974; Block and Reed, 1978). However, this theoretical approach has been transferred to studies investigating longer intervals (from days up to decades) as well. In these studies, differences in passage of time judgments are typically explained by having different numbers of memories available from (a) intervals characterized by a high vs. a low level of routine (Avni-Babad and Ritov, 2003) and (b) from young vs. old age (Wittmann and Lehnhoff, 2005). The differences regarding the availability of memories from recent past are sometimes attributed to a more detailed encoding process of somehow new and interesting information compared to information from stiuations one has experienced regularly. This leads to more memories being available from periods with experiences of novelty (e.g., Tulving and Kroll, 1995; Eagleman and Pariyadath, 2009)^1^. Independently, from the exact mechanism, researchers using the contextual-change-hypothesis when studying the experience of long time-intervals, interpret memories as the source that indicates contextual changes throughout the interval in question. Thus, living a life that provides a multitude of experiences and avoiding routine are considered as remedies to the experience of years fleeting away by researchers, that transfer these deliberations to the experience of long intervals (Bastam, 2018; Sußebach, 2021).

However, to our knowledge, no studies have systematically investigated whether the velocitiy, with which time in the range of several years is judged to have passed, is in fact associated with the number of remarkable events that happened in these years. Given both the notion of non-routine-experiences being encoded and stored better as well as the idea that particularly contextual change-indicating events should be crucial for the experience of time, having experienced more of these notable events should be associated with slower passage of time judgments (POTJs). To examine this, we asked participants to rate their experience of passage of time for the last 5 years (a) before they were presented with a list of outstanding life events, from which participants had to select those that they had experienced within the last 5 years (objective memories, Studies 1a and b) or (b) before they were asked to recall their subjectively most meaningful autobiographical memories from the last 5 years (subjective memories, Study 2).

## Studies 1a and b

These studies were designed to investigate whether the number of particularly meaningful objective life-events that typically indicate change in life (e.g., change of jobs, ending or start of romantic relationships) is associated with passage of time judgments for the interval in which the respective events happened in people's lives. Following the contextual change hypothesis, a larger number of change-indicating events should be associated with slower POTJs for the respective interval. If, however, this association is not present in our data, this suggests that presuming a covert retrieval of memories from particularly meaningful events might not take place when judging the passage of time for multiannual intervals.

The studies were largely alike, differing mainly in adjustments necessary due to the different media used for the surveys: Study 1a was a paper-pencil version, while Study 1b was a replication using an online-survey-tool (www.soscisurvey.com, Leiner, 2014).

## Study 1a

### Methods

#### Participants and Analyses

In April/May 2014, data from 293 participants were collected by using a paper and pencil questionnaire. The participants were mainly recruited in lectures for prospective teachers at the University of Regensburg as well as *via* private networks, which resulted in 89.0% teacher trainees, 9.9% psychology-students and 1.0% other participants. Data from all participants was used for data analysis, however, missing data (e.g., some participants did not fill out questions regarding valence) led to a variation in cases for some analyses. Age ranged from 18 to 34 (*M* = 21.80, *SD* = 2.46), 69.18% of the participants identified as female and 1.71% did not disclose their gender, the rest reported to be male.

All analyses were conducted using the statistical software R (R Core Team, 2021). The associations between the variables were quantified using mixed linear and logistic regression models with the individual responses nested within the subjects (see, Hilbert et al., 2019). For the analyses, the POTJs were associated by modeling them as predictor variable in the regressions with the overall number of events, the number of positive events, and the number of negative events, as dependent variables in the respective models. The regression intercepts were allowed for random variation, as nested within the subjects, and the type-1-error probabilities were corrected for threefold multiple testing *via* the Bonferroni method. The threefold correction was applied, because the four models were all estimated three times in studies 1a, 1b, and 2. The reported p-values are therefore multiplied by three, so that the reference value is still *p* < 0.05.

#### Procedure and Materials

**Figure 4:**
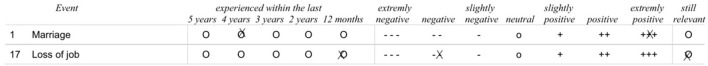
Inductory illustrative examples for the subsequently presented list of life-events.

On the first page, participants were introduced to the questionnaire including a detailed instruction illustrating how to answer the subsequent questionnaire (see Figure 4).

After turning pages, participants were asked to judge their passage of time assessed with one item (“Looking back: how fast did the last 5 years pass by for you personally?”), answers were given on a 7-point Likert scale ranging from “very slow” to “very fast.” Additionally, we asked participants to rate their satisfaction with their current life, their life 3 and 5 years ago as well as with the last 5 years taken together. Answers were given on a seven-point Likert scale ranging from “very slow” to “very fast” and “very unsatisfied” to “very satisfied,” respectively. On the next page, a total of 26 Life Events (e.g., marriage, death of close person, loss of a job; based on Sarason et al., 1978 and Brugha and Cragg, 1990) was presented. Participants had to indicate each event they had experienced in the last 5 years by selecting the according year (radio buttons to select between “12 months” up to “5 years”) and indicate the valence on another 7-point Likert scale (ranging from “extremely negative” to “extremely positive”) as well as whether it was still a matter for the person at the time taking part in the survey (“current relevance”).

After turning pages, participants found two sets of spare lines for (a) adding events that had happened more than once and (b) adding personal events, which had not been covered by the list presented on the previous page. Ultimately, the study was completed by reporting demographic information (age, gender, occupation, and education), five items covering the ease of recall (e.g., “How easy did you find recalling personal events?”) and an open question offering space for any remarks regarding the study.

#### Results and Discussion

Participants judged the 5 years to have passed *M* = 5.62 (*SD* = 1.00) and selected *M* = 7.88 (*SD* = 3.31) events presented on the list. 26.5% were considered as negative (ratings ranging from 1 = very negative to 3 = slightly negative), 66.8% as positive (5 = slightly positive to 7 = very positive), the rest as neutral (4 = neutral). The reported events were *M* = 2.30 (*SD* = 0.68) years in the past. The POTJs were not significantly associated with neither the overall number of objective events reported (γ = −0.03; *p* = 0.85) nor with the number of events reported as positive (γ = −0.05; *p* = 0.30) or negative (γ = 0.02; *p* = 1). Additionally, we analyzed whether the variance of the years since the reported events was related with the POTJs, but found no association between the two variables (γ = 0.01; *p* = 1). All analyses codes and data can be accessed at https://osf.io/7z3yj/?view_only=db7f67c245354c4ba529c36cad0f3259. Given that the events presented on the list are likely to have a significant impact on people's lives and thus indicate change, and given the assumptions of the contextual change hypothesis (Block and Reed, 1978), a higher number of such events should be associated with slower POTJ's. However, the results from this study do not support this prediction.

## Study 1b

### Methods

#### Participants

In January/February 2015, 263 participants filled out the online-version of the study on SoSciSurvey (Leiner, 2014). Participants were recruited through private and university email distribution as well as *via* social media. Age ranged from 18 to 72 (*M* = 28.45, *SD* = 10.64), 69.11% of the participants identified as female, the rest of the sample as male. 50.2% of the participants were students, 47.1% working population, and 2.7% spread among other categories such as job-seeking, attending school or in retirement.

#### Procedure and Materials

The procedure and materials were the same as used in Study 1a with some methodical improvements enabled by and some adjustments necessary due to using the online-platform. After a short introduction, participants were instructed how to answer the subsequent questions. Then, participants judged the passage of the last 5 years as well as their well-being. The Life Events subsequently presented were displayed on separate Pages for each event. Once people indicated that they had experienced the respective event throughout the last 5 years, the information regarding the year of the event (radio buttons ranging from “last 12 months” to “5 years”), the valence (7-point Likert scale ranging from “extremely negative” to “extremely positive”) as well as the current relevance were inquired on the next page^2^. Finally, the participants were asked, whether the same event had taken place more than once during the last 5 years, allowing to specify additional events of the same nature on subsequent pages after selecting “yes” or continuing to the next event by selecting “no.” After having answered the questions regarding all 26 events, participants had the option to report further personal events, which had happened throughout the last 5 years. Then, the ease of recall was enquired before a number of additional questionnaires for research questions addressed for a different project were presented^2^. The survey ended with collecting demographical data and an open question for potential remarks. The statistical analyses were conducted analogously to Study 1a.

#### Results and Discussion

In the online-version, the 5 year interval was judged to have passed with *M* = 5.39 (*SD* = 1.21) and participants reported to have experienced *M* = 9.70 (*SD* = 3.71) of the events from the list. 32.0% of the events were rated as negative, 60.2% as positive, the rest as neutral. The reported events were *M* = 2.47 (*SD* = 0.70) years in the past. The POTJs were not significantly associated with neither the overall number of objective events experienced (γ = −0.03; *p* = 0.46) nor with the number of events rated as positive (γ = −0.05; *p* = 0.14), as negative (γ = 0.03; *p* = 1), or the variance of the ages of the events (γ = 0.07; *p* = 1), thus replicating the findings of the paper and pencil version (Study 1a).

## Study 2

Studies 1a and b investigated whether POTJs are associated with the number of experienced objective events. In these studies, participants were provided with a list of experiences that are likely to be memorable as well as indicative for changes in the participants' lives. However, the items presented are limited and do not necessarily depict the events that actually were the most important and change-indicating for individuals. To address this limitation, we also investigated the association of POTJs with the number of subjectively meaningful memories from the last 5 years. These were inquired in a free-recall paradigm where participants were asked to report as many personally meaningful autobiographical memories as they could spontaneously recall. Following memory based approaches, such as storage size metaphoar and contextual change hypothesis, a higher number of these memories should be associated with slower POTJs for the respective interval. Failing to detect such an association might suggest that a covert retrieval of important memories, which supposedly affects POTJs for multiannual intervals, cannot be presumed.

### Methods

#### Participants and Analyses

Between August and December 2015, 262 persons^3^ completed a questionnaire, in which they reported a POTJ before activating subjective memories. The study was carried out on SoSciSurvey (Leiner, 2014) as well and participants were recruited *via* the website of the German version of Psychology Today (“Psychologie Heute”), the website of our research institute, and private sources. In this sample, age ranged from 14 to 66 (*M* = 32.26, *SD* = 11.65), 74.0% identified themselves as female, 20.2% as male, the rest did not disclose its gender. 31.7% were students, 47.8% employees and 9.8% self-employed, 27.6% spread among other options (e.g., vocational training, household, retired; the selection of multiple options was possible).

As in Studies 1a and 1b, regression models were used to relate the POTJs as predictor variable for the overall number of events, the number of positive events, and the number of negative events, which served as dependent variables in the respective models. Because in this study, the individuals did not select from a pre-defined set of events but reported varying numbers of their individual memories, mixed regression models could not be applied. Therefore, the number of overall, positive, and negative events as well as the mean vanlence (all per subject) served as dependent variables in ordinary least squares regressions. Again, the type-1-error probabilities were corrected for threefold multiple testing *via* the Bonferroni method, due to the three times, each model was estimated, namely in studies 1a and 1b, and 2, and the reported *p*-values therefore multiplied by three.

#### Procedure and Materials

Participants started by filling out the Satisfaction With Life Scale (Glaesmer et al., 2011) and the Positive and Negative Affect Schedule (Krohne et al., 1996) and then judged the Passage of Time for the last 5 years as well as for the last year (7-point Likert scales ranging from “very slow” to “very fast”). Then they were asked to remember as many important autobiographical events from the past 5 years as were spontaneously available, no time limit was given. Each event was entered on a separate page, which depicted an input line allowing a short description of the event. Additionally, each page offered a check box, which could be ticked once the participant wanted to end the input-segment after running out of important autobiographical memories. Events perceived as positive and negative were asked for in separate blocks to avoid state-dependent memory effects (see, e.g., Bower, 1981), the order of these blocks was counterbalanced. Next, each event entered was separately presented to the participants with added questions regarding valence and the time the event took place as well-some other information (importance, subjectively experienced impact on the subsequent life, overcoming of negative events). Finally, demographical information was collected.

#### Results and Discussion

Participants in this study rated the 5 year interval as having passed by with *M* = 5.46 (*SD* = 1.23) and recalled an average of *M* = 7.16 (*SD* = 3.71) subjectively meaningful memories, that were *M* = 2.45 (*SD* = 0.79) years in the past. 30.2% of the events were rated as negative, 57.7% as positive, the rest as neutral. The POTJs were neither related to the overall number of objective events reported (γ = 0.00; *p* = 1) nor to the number of events rated as negative (γ = −0.06; *p* = 1), as positive (γ = 0.10; *p* = 1) or to the variance of the ages of the events (γ = 0.12; *p* = 0.22). This result suggests that neither the number nor the temporal cluster of subjectively meaningful events that someone experienced in a given interval is associated with the velocity, with which the according interval is perceived.

## General Results and Discussion

**Figure 5:**
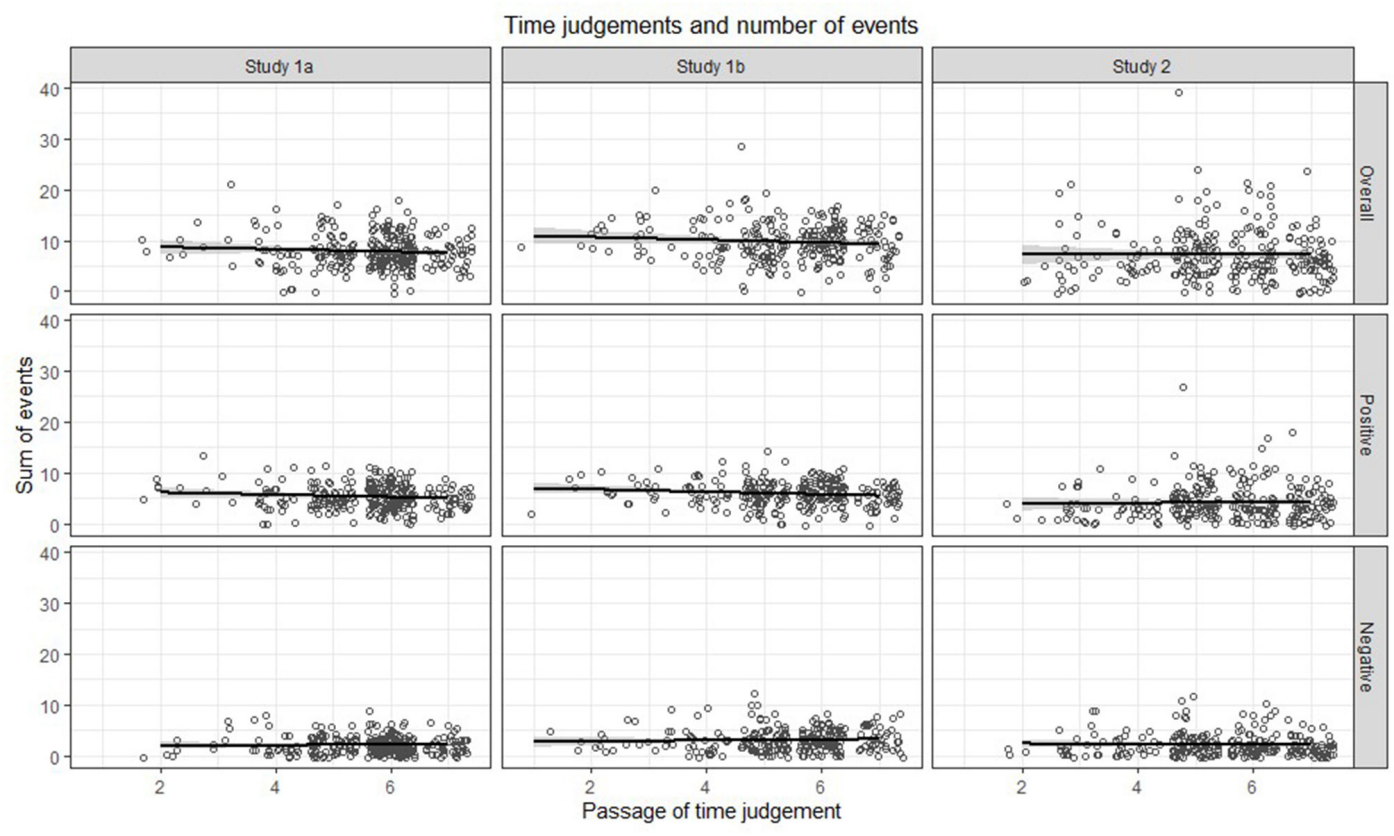
Associations between the total number of events/memories as well as the number of positive and nevative events/memories with Passage of Time Judgments for the last 5 years.

In data from three surveys, we found no evidence for an association between spontaneously given POTJs for the last 5 years and the number or the dispersion of outstanding personal life events that happened during these years (see Figure 5). Given that each sample is large enough to provide a power higher than 95% to detect a small effect of *r*_*P*_ = 0.2^4^ (based on calculations in G^*^Power 3.1.9.7, Faul et al., 2009) and considering that the presented mixed-effect models in Studies 1a and 1b are statistically far more powerful than correlations based on sum scores, it seems likely that an unintended or covert retrieval of meaningful life events as basis for these judgments can be excluded.

Such a covert retrieval has been discussed regarding memory-based approaches and *duration judgments* for intervals in the range of seconds to few minutes (Block and Reed, 1978). In fact, for these short intervals, a covert retrieval seems plausible: Due to the immediacy between encoding and the judging of duration, the information from the interval in question is likely to be still easily accessible.

However, the idea of a covert retrieval has been implicitly transferred to studies investigating passage of time judgments for long intervals, for example, in Wittmann and Lehnhoff's (2005) influential study, differences in POTJs for 10 years are attributed to “variations of activities, life events” (p. 933), directly referring to the contextual change hypothesis. Since data regarding these autobiographical events (or memories thereof) or other changes are not assessed in these studies, the covert retrieval of these memories seems to be premised. Given our results, however, it might be hasty to explain differences in POTJs for multiannual intervals without these memories and/or life-events actually having been assessed in the regarding study.

Two other studies covering longer intervals, where this idea has been implicitly presupposed as well, can be found in Avni-Babad and Ritov (2003): Here, vacationers were asked to split their holidays in three parts and to compare the experienced duration of these (Study 5). Moreover, inhabitants of a kibbutz (a rural community in Israel with inhabitants collaboratively living and working together) were asked to judge the passage of time for their regular job and one they did work exceptionally (Study 6). Results showed that duration was judged as shorter for the last part of the holiday and passage of time was judged faster for the regular job. These findings were interpreted as a consequence of “fewer stimuli to remember” (p. 549) due to higher levels of routine. Again, this would imply that a (potentially covert) retrieval has to take place when judging the passage of time. Although this retrieval was not an explicit part of the design, in this case, the instructions direct their participants attention to reasonable intervals (1 or 2 days of a 3–4 day-vacation) and concrete experiences (the regular and exceptional job), potentially directly inducing a retrieval of memories. That is, when asking explicitly for the experience of time of certain days/situations, memories from these instances could have been activated and a comparison of spontaneously recalled memories between the respective timeframes might have led to the different judgments. Summed up, both the presumed activation of memories and the direct comparison between different intervals are likely to have affected the reported experience of time since both processes are potentially crucial for these judgments. Evidence from our previous study supports this interpretation, since we were able to show that activating a crucial number of autobiographical memories before judging the passage of time led to judgments of these years as having passed slower compared to having activated very little or no memories (Kosak et al., 2019).

In the light of this prior finding, it seems that memories still play a crucial role for the perceived velocity of years passing. Presumably, however, spontaneous judgments of the passage of time for multiannual intervals are not systematically affected by the mere amount of experienced remarkable life-events as long as memories from these are not directly activated prior to judging (Kosak et al., 2019). Other factors, such as perceived stress (e.g., Janssen, 2017) or the ratio of time-units (in this case of years) to life-time (as discussed in Block et al., 1998) might be important too when trying to understand spontaneous judgments of time for multiannual intervals.

However, we have to acknowledge that interpreting null-findings is generally a challenging task because these can result from a variety of causes. Nevertheless, the fact that we found a consistent pattern in three independent datasets using highly powered analyses gives the results credibility. But of course, our approach comes with methodological limitations that might restrain the generalizabilty of the presented findings. First, we investigated only one particular interval, namely 5 years. Although it seems likely that this null-result is transferable to other multiannual intervals, it is possible that investigating intervals in the range of days or even several months (both still “long” compared to most of the research conducted in this field) with this approach leads to different results. The content of such intervals might be more comprehensible and/or salient memories from the past might subconsciously affect the experience of time since, in such a case, these are less far in the past at the moment of judging the passage of time. Regarding intervals of several days, the studies presented by Avni-Babad and Ritov (2003) might be interpreted as preliminary evidence for such an assumption.

Second, the list of events used in Studies 1a and 1b covered only a limited number of life-events and thus might not have depicted all potentially crucial events. However, we tried to fill this blind spot by applying the free-recall-paradigm in Study 2, which did not reveal different results. However, Study 2 has its own limitations: Although, we consciously decided to set no time limit for the recall in order to avoid any pressure for our participants, we can not exclude that this has led to an inflation of recalled memories. Possibly, a limitation of time for the recall-stage of the study would lead to a retrieval of fewer memories and this reduced number might be a better representation of potentially change-indicating events.

Despite these limitations, the current studies are an important first approach in trying to verify the common presumption that the number of important and/or change-indicating memories affect the experienced passage of longer time-intervals. Our results provide no support for this presumption. This suggests that explaining the experience of time for multiannual intervals by applying insights from memory-based theories, which have been validated only with duration judgments for short intervals, is potentially premature.

After all, when investigating POTJs for long intervals, it might be important to incorporate a recent finding: Lee and Janssen (2019) were able to show that personal believes about the passage of time affect the judgments of these. Given that a majority of people in industrialized countries believes that time goes faster with aging (Lee and Janssen, 2019), presumably with different underlying theories explaining this phenomenon, spontaneous POTJs regarding long intervals might also reflect conclusions individuals draw from their personal believes about the passage of time. Simply put, spontaneous and decontextualized POTJs might—from a researcher's perspective—evoke relatively arbitrary ratings, which do not necessarily reflect an actual experience of time, but rather believes people have about the passage of time. A study investigating POTJs and its relation to laypersons theories and concepts regarding the passage of time would be necessary to clarify these assumptions. For the time being, we must conclude that a covert retrieval of remarkable, change-indicating memories seems not to be a decisive mechanism explaining differences in Passage of Time Judgments for multiannual intervals.

## Data Availability Statement

The data as well as the analyses reported in this article are available under https://osf.io/7z3yj/.

## Ethics Statement

Ethical approval was not provided for this study on human participants because the according study is in line with the declaration of Helsinki. Studies like ours do not need an additional approval by an Ethics Commitee in Germany (see https://www.dfg.de/foerderung/faq/geistes_sozialwissenschaften/). Written informed consent from the participants' legal guardian/next of kin was not required to participate in this study in accordance with the national legislation and the institutional requirements.

## Author Contributions

FK designed and conducted the studies and wrote the draft of the manuscript. SH contributed the analyses, wrote the according sections, and revised the manuscript. All authors contributed to the article and approved the submitted version.

## Conflict of Interest

The authors declare that the research was conducted in the absence of any commercial or financial relationships that could be construed as a potential conflict of interest.

## Publisher's Note

All claims expressed in this article are solely those of the authors and do not necessarily represent those of their affiliated organizations, or those of the publisher, the editors and the reviewers. Any product that may be evaluated in this article, or claim that may be made by its manufacturer, is not guaranteed or endorsed by the publisher.

## Footnotes

^1^Research covering the so-called reminiscence bump, i.e., the phenomenon that most important autobiographical memories from elderly stem from their youth/young adulthood, makes use of a number of additional approaches explaining the lack of memories from recent years (see Ece and Gülgöz, 2014, for an overview).

^2^Additionally, the experience of control regarding the respective event was surveyed here as well, using one item asking “Did you have the impression, that you were able to control the situation?”, answered on another 7-point Likert scale ranging from “The situation was not controlable at all” to “The situation was very controllable.” These measures were used for a different research question, addressed in Kugler et al. (2021).

^3^This is a subsample taken from Kosak et al. (2019), where about the same number of persons did activate their experiences before judging the passage of time.

^4^Pooling the samples leads to a correlation of *r*_*P*_ (816) = −0.04, *p* = 0.14 between the number of events/memories and the POTJs. This implies a power of 99.99% to detect an effect of *r*_*P*_ = 2. Note that due to the varying numbers of memories per participant in Study 2, no multilevel analysis was conducted for the pooled sample.
